# Amorphous MoS_x_O_y_/*h*-BN_x_O_y_ Nanohybrids: Synthesis and Dye Photodegradation

**DOI:** 10.3390/nano11123232

**Published:** 2021-11-28

**Authors:** Andrei T. Matveev, Anton S. Konopatsky, Denis V. Leybo, Ilia N. Volkov, Andrey M. Kovalskii, Liubov A. Varlamova, Pavel B. Sorokin, Xiaosheng Fang, Sergei A. Kulinich, Dmitry V. Shtansky

**Affiliations:** 1 Laboratory of Inorganic Nanomaterials, National University of Science and Technology “MISIS”, Leninskiy Prospect 4, 119049 Moscow, Russia; konopatskiy@misis.ru (A.S.K.); leybo.dv@misis.ru (D.V.L.); ilia.volkov@misis.ru (I.N.V.); a.kovalskiy@misis.ru (A.M.K.); varlamova.la@misis.ru (L.A.V.); pbsorokin@misis.ru (P.B.S.); shtansky@shs.misis.ru (D.V.S.); 2 Department of Materials Science, Fudan University, Shanghai 200433, China; xshfang@fudan.edu.cn; 3 Research Institute of Science and Technology, Tokai University, Hiratsuka 259-1292, Kanagawa, Japan; 4 School of Engineering, Far Eastern Federal University, 690041 Vladivostok, Russia

**Keywords:** molybdenum sulfide, hexagonal BN, MoS_x_O_y_/BN_x_O_y_ nanohybrids, photocatalytic degradation of methylene blue, DFT analysis

## Abstract

Molybdenum sulfide is a very promising catalyst for the photodegradation of organic pollutants in water. Its photocatalytic activity arises from unsaturated sulfur bonds, and it increases with the introduction of structural defects and/or oxygen substitutions. Amorphous molybdenum sulfide (*a*-MoS_x_O_y_) with oxygen substitutions has many active sites, which create favorable conditions for enhanced catalytic activity. Here we present a new approach to the synthesis of *a*-MoS_x_O_y_ and demonstrate its high activity in the photodegradation of the dye methylene blue (MB). The MoS_x_O_y_ was deposited on hexagonal boron oxynitride (*h*-BNO) nanoflakes by reacting *h*-BNO, MoCl_5_, and H_2_S in dimethylformamide (DMF) at 250 °C. Both X-ray diffraction analysis and high-resolution TEM show the absence of crystalline order in *a*-MoS_x_O_y_. Based on the results of Raman and X-ray photoelectron spectroscopy, as well as analysis by the density functional theory (DFT) method, a chain structure of *a*-MoS_x_O_y_ was proposed, consisting of MoS_3_ clusters with partial substitution of sulfur by oxygen. When a third of the sulfur atoms are replaced with oxygen, the band gap of *a*-MoS_x_O_y_ is approximately 1.36 eV, and the valence and conduction bands are 0.74 eV and −0.62 eV, respectively (relative to a standard hydrogen electrode), which satisfies the conditions of photoinduced splitting of water. When illuminated with a mercury lamp, *a*-MoS_x_O_y_/*h*-BN_x_O_y_ nanohybrids have a specific mass activity in MB photodegradation of approximately 5.51 mmol g^−1^ h^−1^, which is at least four times higher than so far reported values for nonmetal catalysts. The photocatalyst has been shown to be very stable and can be reused.

## 1. Introduction

Overall, solar dissociation of water can be expressed by two half reactions: the hydrogen evolution reaction (HER) and the oxygen evolution reaction (OER). HER is being thoroughly investigated as a potential green energy source that could reduce the environmental crisis caused by carbon dioxide emissions from the use of hydrocarbons for energy production. Pollution of the aquatic environment by industrial emissions, as well as wastes from the chemical, pharmaceutical and cosmetic industries lead to catastrophic consequences for the flora and fauna [1]. OER not only generates oxygen gas, but is also used to purify and sterilize water through the generation of reactive oxygen species. Photocatalytic degradation by sunlight using safe catalytic materials is a green technology with minimal environmental impact. To increase the efficiency of solar energy harvesting new materials, their microstructure, chemical composition, electronic band structure, as well as heterojunction with other materials and architecture of photoelectrochemical cells are being thoroughly studied [2,3,4].

In recent years, molybdenum disulfide has been actively studied as a catalyst for photo- and electrochemical processes, such as HER [5,6]. Both theoretical and experimental studies show that its activity in HER arises from the presence of unsaturated sulfur at the edges [5,6,7]. Thus, decreasing the size of MoS_2_ clusters is a promising strategy for increasing catalyst efficiency. In addition to edge defects, the disordered crystal structure provides many active sites with complex atomic configurations that increase activity [6]. The high catalytic activity of amorphous molybdenum sulfide has been proven, which is explained by the high density of active centers in the disordered structure [7,8,9,10]. Another widely used approach is to adjust local charge distribution by atomic substitutions [11]. In the case of molybdenum sulfide, it was shown that the incorporation of oxygen in the MoS_2_ structure leads to a noticeable increase in the HER activity [8,10]. An increased reactivity of hydrogen evolution was observed in oxygen-incorporated MoS_2_-based nanostructures, obtained as a result of the interfacial reaction between MoS_2_ and MoO_3_ [12]. With the use of MoS_2_/WS_2_ heterostructures, the possibility of hydrogen formation in seawater under sunlight has recently been shown [13]. Thus, structural disorder and oxygen substitutions in molybdenum sulfide, as well as the adjacent of its energy bands due to interaction with other materials, are a prerequisite for high catalytic activity in HER.

In addition to its high activity in HER, molybdenum sulfide also has a high activity in the OER. Computational analysis of the positions of the conduction and valence band edges of single-layer MoS_2_ predicted that it is a potential photocatalyst for water splitting [14,15]. Shortly thereafter, it was confirmed that MoS_2_ exhibits excellent OER performance in acidic solution, the 1T-MoS_2_ polymorph exhibits catalytic efficiency comparable to the most well-known IrO_2_ electrocatalyst, and DFT calculations showed that the activity originates from edge sites [16,17,18]. Atomically thin MoS_2_ layers oxidize water when exposed to sunlight in an acidic environment, releasing O_2_ gas [19]. Remarkable OER activity was found in MoS_2_ quantum dots. Theoretical analysis showed that in this case, the vertices are active sites, and sulfur vacancies play an important role in the catalytic activity [20]. Nanostructured molybdenum sulfide appears to be a very promising material for photodegradation of anthropogenic organic waste [21,22,23]. At the same time, it was noted that the photooxidative ability of MoS_2_ is impeded by insufficient generation of ·OH radicals and this obstacle can be overcome by creating heterojunctions [24]. More recently, an increase in overall water splitting was demonstrated through the design of heterogeneous interfaces in CoS/MoS_2_ binary composites [25].

Amorphous molybdenum sulfide (*a*-MoS_x_) films were obtained by various physical deposition methods, such as magnetron sputtering, pulsed laser deposition, and atomic layer deposition [26,27]. These methods are difficult to adapt for film deposition on the surface of dispersed powders. An alternative strategy is to obtain *a*-MoS_x_ films in an aqueous media using a chemical reaction with MoO_3_. For example, *a*-MoS_x_ thin films were prepared at room temperature by electrochemical deposition from aqueous solutions [28,29]. *a*-MoS_3_ powder containing MoS_2_ domains was synthesized by wet chemistry in an acidic aqueous media at room temperature [11]. The hydrothermal process was also used to synthesize *a*-MoS_x_ quantum dots [30]. All these chemical methods were carried out in aqueous media, and the synthesis of molybdenum sulfide occurred due to the reduction of MoO_3_ clusters. This largely determines the direction of chemical transformations and consequently, the local atomic structure of the resulting product.

In this paper, we have developed a new method for the synthesis of *a*-MoS_x_O_y_/*h*-BN_x_O_y_ nanohybrids and studied their activity in the process of photodegradation of methylene blue (MB) as a model organic pollutant. Nanohybrid materials were synthesized by a chemical reaction between *h*-BN_x_O_y_, MoCl_5_ and H_2_S dissolved in dimethylformamide (DMF). The main difference of this method is that the synthesis of molybdenum sulfide proceeds through the reaction of MoCl_5_ with H_2_S, and the MoO_3_ clusters are not involved in the synthesis. This can lead to a different local atomic order compared to the reduction of MoO_3_. The use of liquid media for synthesis provides homogeneous deposition of *a*-MoS_x_O_y_, and a relatively low synthesis temperature ensures its amorphous state. *h*-BN was chosen as a substrate because of its pronounced synergistic properties as an adsorbent-photoactive material [31]. Oxygen-substituted BN (*h*-BN_x_O_y_) was used because of its improved wettability. In addition, the reaction of oxygen defects with sulfur results in the formation of S-doped BN, which itself has impressive photodegradation efficiency even under visible light illumination [32].

Herein, nanohybrids based on amorphous molybdenum oxysulfide (*a*-MoO_x_S_y_) and hexagonal boron nitride (*h*-BN) are studied as a promising photocatalytic material. Both components are non-toxic and chemically stable [33,34]. *h*-BN is very slowly hydrolyzed in water to form boric acid, which has antimicrobial and antibacterial properties. Molybdenum oxysulfide is a narrow bandgap semiconductor, while BN is a wide bandgap semiconductor that maximizes the use of the solar spectrum in both the visible and ultraviolet regions.

## 2. Materials and Methods

### 2.1. Raw Materials

*h*-BN_x_O_y_ nanopowder (Plasmotherm, Moscow, Russia) consisted of nanoflakes with a thickness of several atomic layers and a lateral size of approximately 20 nm. As-received material was dried under vacuum at 200 °C for 3 h to remove absorbed water.

Chemical grade dimethylformamide (DMF, 99.9% purity) was used as the medium for the synthesis of *a*-MoS_x_O_y_/*h*-BN_x_O_y_ nanohybrids. DMF was chosen because of its high ability to dissolve both MoCl_5_ and H_2_S. Among common solvents, DMF has almost the highest solubility for hydrogen sulfide—at 25 °C 1 mol of DMF dissolves 0.12 mol of H_2_S [35]. It has also been reported that the dielectric constant of DMF over 99.8% purity at 25 °C is 37.3 [36], which allows microwave heating to be used instead of conventional heating in a resistive oven. This makes the synthesis process more flexible.

### 2.2. Fabrication of a-MoS_x_O_y_/h-BN_x_O_y_ Nanohybrids

DMF was dried for one week using silica gel, then 50 mL of dried DMF was poured into a thin long tube and hydrogen disulfide was bubbled through the liquid for at least 40 min to ensure complete saturation, which is reached in less than 20 min [31]. Hydrogen disulfide was prepared by heating of a mixture of paraffin (5 g) and sulfur (3 g) at 200 °C using an Ar flow (99.99%) as a transport gas. 0.5 g of MoCl_5_ was dissolved in 20 mL of dried DMF, then 1 mL of this solution with 30 mL of DMF saturated with H_2_S, and 300 mg of dried *h*-BN_x_O_y_ nanopowder were placed in a Teflon vessel in a microwave oven (ETHOS EASY, Milestone S.r.l., Sorisole, Italy) and tightly closed. All manipulations were carried out in an Ar atmosphere. The vessel was subjected to microwave heating to a temperature of 250 °C at a rate of 15 °C/min and held at this temperature for 30 min, after which it was allowed to cool spontaneously. The temperature during the experiment was controlled by a thermocouple immersed in the liquid. This selected synthesis temperature is below the temperature at which the formation of crystalline molybdenum sulfide from MoCl_5_ and H_2_S was observed during the chemical vapor deposition (CVD) process [37]. After synthesis, the liquid turned black and had pH of 8. The resulting product was thoroughly washed, first five times in distilled water, and then several times in ethanol using an ultrasound bath. After centrifugation, the powder was dried under vacuum at 200 °C. The powder thus obtained was gray in color.

### 2.3. Material Characterization

Chemical analysis of *a*-MoS_x_O_y_ was performed using an iCAP 6300 inductively coupled plasma atomic emission spectroscope (Thermo Fisher Scientific Inc., Waltham, MA, USA). The microstructure and distribution of elements were studied using scanning transmission electron microscopy (STEM) and high-angle annular dark-field imaging (HAADF) in combination with energy dispersive X-ray spectroscopy (EDXS) using a Tecnai Osiris S/TEM 200 kV microscope (FEI, Hillsboro, OR, USA). The phase composition was determined on a SmartLab diffractometer (Rigaku, Tokyo, Japan) using Cu-Kα radiation and a graphite monochromator. X-ray diffraction (XRD) patterns were recorded in the symmetrical mode and analyzed using PDXL software (Rigaku). Fourier-transform infrared (FTIR) spectra were recorded on powder samples using a Vertex 70v vacuum spectrometer (Bruker, Billerica, MA, USA) in the range of 400–4000 cm^−1^ using a partial internal reflection device. Raman spectra were collected using a DXR3 Raman spectrometer (Thermo Fisher Scientific Inc.) with a wavelength of 532 nm. Surface chemical bonds were analyzed by means of X-ray photoelectron spectroscopy (XPS) using a Versa Probe III (PHI, 18725 Lake Drive East, Chanhassen, MN 55317, USA) equipped with a monochromatic Al Kα X-ray source (hν =1486.6 eV). The binding energy values were calibrated using the C 1s line (284.8 eV) derived from the adventitious carbon. The spectra were processed in the Casa XPS.

### 2.4. Photocatalytic Measurements

For photocatalytic measurements, 6 mg of the synthesized MoS_x_O_y_/*h*-BN_x_O_y_ nanohybrids and 1 mg of methylene blue (MB) were dissolved in 50 mL of distilled water in quartz glass and dispersed in an ultrasound bath for 5 min. pH of the solution was neutral. Then the mixture was stirred with a magnetic stirrer for 30 min until the sorption-desorption equilibrium was reached. All these manipulations were carried out in the dark. The resulting solution was illuminated with UV radiation for 60 min using a mercury lamp (145 W) with constant stirring. Photocatalytic degradation was analyzed by measuring the intensity of the ultraviolet-visible (UV-vis) absorption spectra recorded on a UVmini-1240 spectrophotometer (Shimadzu). During irradiation, 1 mL of the filtered solution was taken after 5, 15, 30 and 60 min. For comparison, the photocatalytic degradation of the initial *h*-BN_x_O_y_ nanoparticles (without MoS_x_O_y_) was studied using the same protocol. The specific catalyst mass activity was calculated as the number of moles of degraded MB divided by the weight of catalyst (calculated for the weight of *a*-MoS_x_O_y_) per hour. The stability and reusability of the photocatalysts were evaluated using three consequent photocatalytic degradation tests. After each test, the catalyst was collected by centrifugation and placed in fresh dye solution without washing for retesting.

### 2.5. DFT Analysis

Density functional theory (DFT) calculations were performed in the Viena Ab initio Simulation Package (VASP, version 5.4.4) with the Perdew–Burke-Ernzerhof (PBE) functional and a plane wave cutoff of 400 eV [38,39,40]. 

## 3. Results

### 3.1. Characterization of BNO and a-MoS_x_O_y_/h-BN_x_O_y_ Materials

According to energy dispersive X-ray spectroscopy (EDXS) analysis, the *h*-BN_x_O_y_ contains ~10 at.% of oxygen. ICP analysis of *a*-MoS_x_O_y_ shows 3.06 wt.% of Mo and 2.01 wt. % of S, which corresponds to a Mo/S ratio of approximately 0.51.

**Figure 1:**
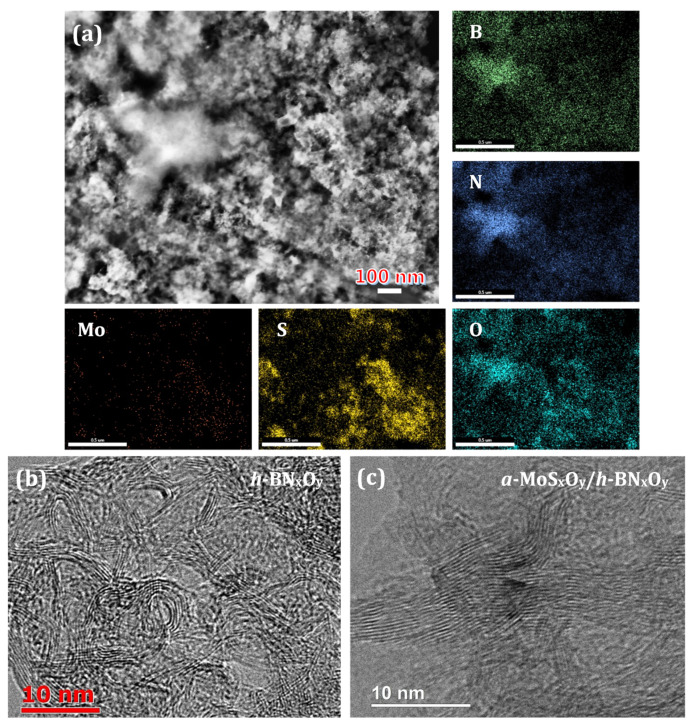
HAADF-STEM image with corresponding EDXS elemental maps of the as-synthesized *a*-MoS_x_O_y_/*h*-BN_x_O_y_ nanohybrids (**a**). High-resolution TEM micrographs of the pristine *h*-BN_x_O_y_ (**b**) and as-synthesized *a*-MoS_x_O_y_/*h*-BN_x_O_y_ nanohybrids (**c**).

**Figure 2:**
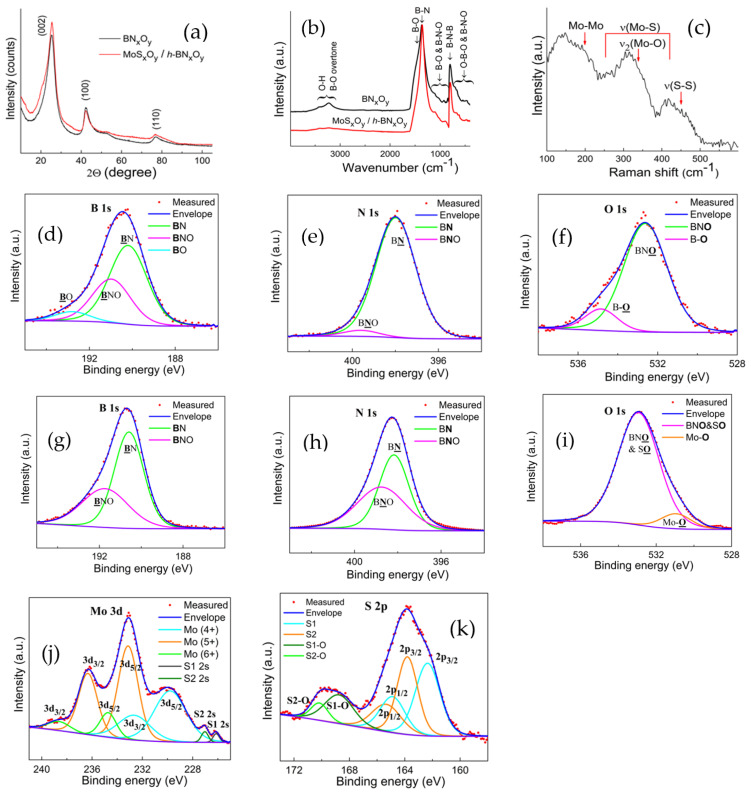
XRD patterns (**a**) and FTIR (**b**), Raman (**c**), and XPS (**d**–**k**) spectra of *h*-BN_x_O_y_ and *a*-MoS_x_O_y_/*h*-BN_x_O_y_ materials. *h*-BN_x_O_y_—(**a**,**b**,**d**–**f**), *a*-MoS_x_O_y_/*h*-BN_x_O_y_—(**a**–**c**,**g**–**k**).

**Figure 3:**
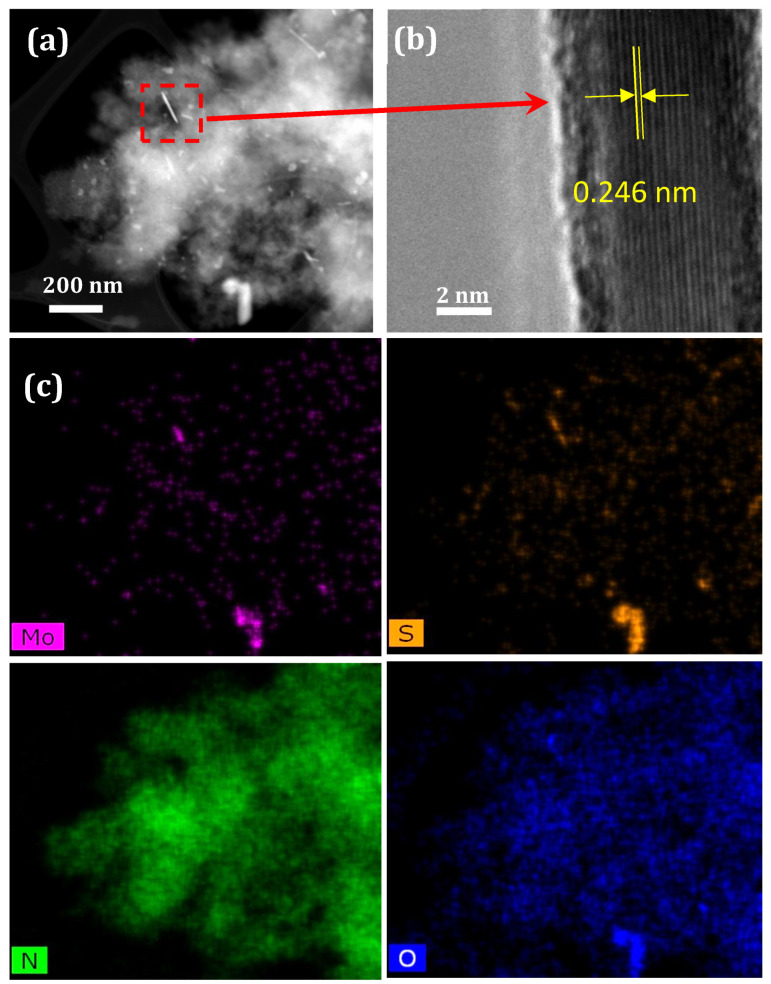
HAADF-STEM (**a**) and HRTEM (**b**) images with corresponding EDXS elemental maps (**c**) of *a*-MoS_x_O_y_/*h*-BN_x_O_y_ nanohybrids after annealing in vacuum at 600 °C for 1 h.

A HAADF-STEM image with the corresponding EDXS elemental maps of *a*-MoS_x_O_y_/*h*-BN_x_O_y_ nanohybrids is shown in Figure 1a. The distributions of boron, nitrogen, and oxygen correlate with each other, indicating the presence of *h*-BN_x_O_y_ phase. Areas rich in molybdenum, sulfur and oxygen also overlap, implying their chemical bonding. The high resolution transmission electron microscopy (HRTEM) image confirms that the initial *h*-BN_x_O_y_ consists of curled flakes with an average thickness of several atomic layers and a length of ~20 nm (Figure 1b). It can be seen that after synthesis, the full width at the half maximum (FWHM) of the XRD peak increases slightly, which indicates a decrease in the average size of *h*-BN_x_O_y_ nanocrystals (Figure 2a). We believe that this is due to hydroxylation and partial etching of *h*-BN_x_O_y_ nanocrystals in the alkaline synthesis medium (pH 8) at 250 °C [41]. It should be noted that no diffraction peaks from molybdenum sulfide are seen in the XRD pattern. According to the ICP AES analysis, the concentration of molybdenum sulfide is 5 wt.%. The crystalline molybdenum sulfide in such an amount should be detected by XRD. The absence of reflection from molybdenum sulfide suggests an amorphous state. For proof, the nanohybrids were vacuum annealed at 600 °C for 1 h. The HAADF-STEM image and the corresponding EDXS elemental maps are presented in Figure 3. After annealing S, Mo, and O are concentrated in separate nanoparticles due to segregations. Rod-like precipitates observed in Figure 3a, are identified as crystalline nanoparticles with a characteristic *d* spacing of 0.246 nm, typical of the MoS_2_ phase (Figure 3b).

The presence of oxygen-substituted *h*-BN_x_O_y_ phase (as evidenced from the EDXS analysis) was also confirmed by FTIR spectroscopy. The FTIR spectrum of initial *h*-BN_x_O_y_ nanopowder exhibits strong peaks at ~1360 and ~798 cm^−1^, corresponding to the stretching and bending vibrations of B–N bonds in the B–N–B network [42] (Figure 2b). In addition, weak bands in the ranges of ~450–750 cm^−1^ and 860–1130 cm^−1^ are observed, which are associated with B-O vibrations. Two broad bands of moderate intensities in the range of ~3070–3500 cm^−1^ are attributed to the overtones of B–O and O–H vibrations. After the synthesis of *a*-MoS_x_O_y_/*h*-BN_x_O_y_ nanohybrids, the FTIR spectrum changed noticeably. The bands in the high frequency range (~3070–3500 cm^−1^) have almost disappeared, and the shoulder at ~1400 cm^−1^ is not visible. A small band at about 700 cm^−1^ also became less pronounced. All these changes indicate that boron oxide and terminated hydroxyl groups formed as a result of partial hydrolysis of the *h*-BN_x_O_y_ phase are reduced. However, some small peaks in the ranges of 1100–850 cm^−1^ and 700–400 cm^−1^ are still visible. We believe that these peaks are associated with the oxygen-substituted *h*-BN_x_O_y_ phase.

XRD analysis suggested that the molybdenum sulfide was in an amorphous state. The absence of crystalline MoS_2_ was also confirmed by Raman spectroscopy. Raman spectrum of the *a*-MoS_x_O_y_/*h*-BN_x_O_y_ nanohybrids is shown in Figure 2c. Three broad bands are observed in the ranges of 100–250 cm^−1^, 250–380 cm^−1^, and 380–500 cm^−1^. The low peak of the Raman shift can be ascribed to Mo–Mo vibrations [43,44,45]. The second band (250–380 cm^−1^) is associated with coupled *ν*(Mo–S) [43,44] and terminal Mo-S_t_ vibrations [43,44,45,46,47]. Mo–O bonds have also been reported in the range of 300–340 cm^−1^ [48]. The maximum of the third band (380–500 cm^−1^) is located at ~415 cm^−1^, which is close to the A_1g_ vibrations in MoS_2_ observed at 407–411 cm^−1^. However, this band should be accompanied by the E^1^_2g_ feature at ~380 cm^−1^ [12,47], which is not visible in the spectrum. Therefore, we assume that there is no hexagonal MoS_2_ in the sample. Maximum in the range of 380–500 cm^−1^ can be attributed to the *ν*(Mo_3_-*µ*S) vibrations [9]. However, the terminal and bridging S-S bonds in the Mo_3_S_x_ complexes have vibrations at 515, 546, 552 cm^−1^ [44,45] which are not visible in our spectrum, which indicates the absence of triangular complexes of molybdenum sulfides. Thus, from the Raman spectroscopy analysis it can be concluded that the material consists of molybdenum oxysulfide clusters [Mo_x_S_y_O_z_], but, apparently, without apical sulfur. The large width of bands indicates that the clusters have a high degree of chemical nonuniformity both in stoichiometry and in the coordination environment of the elements. It should be noted, that in the case of an amorphous material, assignment of the observed Raman shifts is a challenge, since the selection rules based on crystal symmetry are weakened. In addition, some of the bands observed in the crystalline material may disappear, while new bands may appear, which further complicates the analysis [47,49]. Therefore, for a deeper insight in the chemical bonding, XPS method was employed.

**Figure 4:**
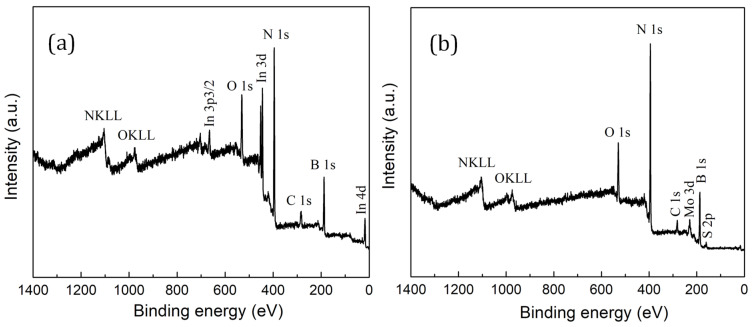
Survey spectra of *h*-BN_x_O_y_ (**a**) and *a*-MoS_x_O_y_/*h*-BN_x_O_y_ materials (**b**).

The survey XPS spectra of *h*-BN_x_O_y_ and *a*-MoS_x_O_y_/*h*-BN_x_O_y_ materials placed on an In substrate are shown in Figure 4. In addition to the small XPS C 1s peak from adsorbed carbonaceous species, the *h*-BN_x_O_y_ XPS spectrum shows O 1s, N 1s, and B 1s peaks as well as In substrate peaks. The XPS spectrum of *a*-MoS_x_O_y_/*h*-BN_x_O_y_ nanohybrids additionally contains Mo and S. Peaks of Cl are not observed.

High-resolution XPS spectra of the *h*-BN_x_O_y_ and *a*-MoS_x_O_y_/*h*-BN_x_O_y_ materials are depicted in Figure 2d–k. The XPS B 1s spectrum of *h*-BN_x_O_y_ was deconvoluted into three components: B–O (192.8 eV), BNO (191 eV), and BN (190.2 eV) [50]. The XPS N 1s peak (Figure 2e) was approximated by two peaks at ~398 eV (BN) and at ~399 eV (BNO). Two peaks observed in the XPS O 1s spectrum at ~532.6 eV and ~534.8 eV were ascribed to the BNO and BO phases, respectively (Figure 2f). Thus, the results of XPS analysis of the *h*-BN_x_O_y_ sample are in good agreement with the IR spectra (Figure 2b) and confirm the presence of BN (main component), BNO (secondary component) and BO (minor phase).

In *a*-MoS_x_O_y_/*h*-BN_x_O_y_ nanohybrids, the XPS B 1s peak (Figure 2g) was deconvoluted into two components located at 190.6 eV (BN) and 191.7 eV (BNO). Boron oxide is not observed, and the BNO peak has slightly shifted towards a higher binding energy as compared to the peak of the *h*-BN_x_O_y_ material. It can be assumed that boron oxide reacted with boron oxynitride phase, which led to an increase in its doping with oxygen. Nitrogen in the XPS N 1s spectrum appears in two chemical environments with binding energies of 398.8 eV and 398.2 eV, which corresponds to the BNO and BN phase, respectively (Figure 2h). Deconvolution of the XPS O 1s spectrum shows two components at 531 eV (Mo–O) and at 532.9 eV (BNO). Since the binding energy of the S–O bond also has a peak at 531 eV, it is difficult to distinguish between the BNO and SO bonds. Figure 2i also shows that the B–O bond disappeared and a new Mo–O bond appeared (Table 1).

XPS analysis also showed that Mo and S are present in various types of coordination within an amorphous MoO_x_S_y_ phase. The XPS Mo 3d spectrum exhibits three strong peaks (Figure 2j). Their best fitting is obtained by deconvolution the spectrum into three overlapping spin-orbit doublets 3d_5/2_ and 3d_3/2_ at (i) 234.8 and 237.9 eV, (ii) 233.1 and 236.2 eV, (iii) (229.8 and 232.9 eV) corresponding to three oxidation states of molybdenum: Mo (6^+^), Mo (5^+^), and Mo (4^+^) (Table 2). The relative content of these states is calculated as 10, 49, and 39%, respectively, which means that the average oxidation state of Mo is (+4.61). The highest oxidation state of Mo (6^+^) is apparently associated with MoO_3_, the formation of which is also revealed by the Mo–O bond in the O 1s spectrum (Figure 2i). The second Mo (3d) doublet at 233,1 and 236, 3 eV, corresponding to Mo (3d_5/2_) and Mo (3d_3/2_), belongs to the Mo (5^+^) state in MoO_x_S_y_. We attribute Mo in the oxidation state (4^+^) to MoS_3_ and not to MoS_2_, because the latter phase is not identified by Raman analysis. In MoS_3_, sulfur is present in at least two states, including the S^2−^ and S_2_^2−^ ligands in various types of coordination [51]. Indeed, two peaks in the XPS S 2s spectrum are observed at 226.1 and 227.0 eV (Figure 2j). We attribute these peaks to the bridging and terminal S_2_^2−^ in Mo–S_3_ clusters. The XPS S 2p spectrum (Figure 2k) also contains two spin-orbit 2p_3/2_ and 2p_1/2_ doublets. The doublet at 162.4 and 164.9 eV originates from the terminal S_2_^2−^ ligands, and the doublet at 163.8 and 165.3 eV is associated with bridging or shared S_2_^2−^ ligands [9,51,52,53]. Also, the doublet at higher BE values can be explained by polysulfide clusters. The remaining high-energy peaks in the XPS S 2p spectrum at 168.8 eV and 170.1 eV are attributed to S–O bonds (Table 3).

### 3.2. Photocatalytic Activity

**Figure 5:**
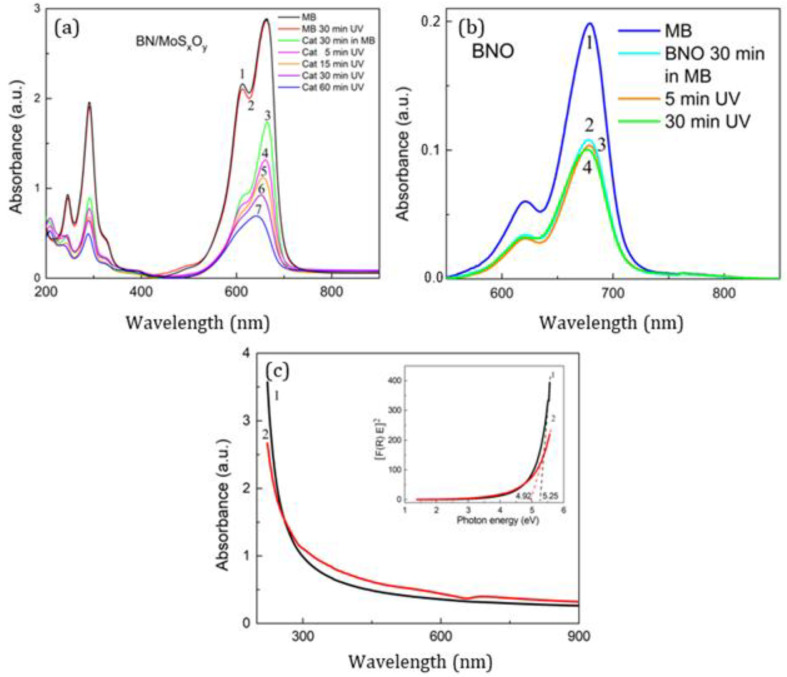
UV-vis absorption spectra of MB water solution (**a**). 1—MB without UV-illumination, 2—MB after illumination for 30 min, 3—MB + *a*-MoS_x_O_y_/*h*-BN_x_O_y_ (denoted as Cat) after 30 min exposure in the dark, 4–7—Cat after illumination for 5, 15, 30, and 60 min. UV-vis absorption spectra of MB + *h*-BN_x_O_y_ suspension (**b**). UV-vis absorption spectra (**c**) of *h*-BN_x_O_y_ (curve 1) and *a*-MoS_x_O_y_/*h*-BN_x_O_y_ (curve 2) nanopowders suspended in methanol. The inset shows the Tauc plot and band gap values for *h*-BN_x_O_y_ (curve 1) and *a*-MoS_x_O_y_/*h*-BN_x_O_y_ (curve 2).

The photocatalytic activity of the *a*-MoS_x_O_y_/*h*-BN_x_O_y_ heterostructures was studied using methylene blue (MB) as a model pollutant. Figure 5a shows the UV-vis absorption spectra of an MB aqueous solution (1 mL MB per 50 mL of water, which corresponds to concentration of 62.5 mmol/L) before UV irradiation (curve 1), after irradiation for 30 min (curve 2), as well as the solution of MB + *a*-MoS_x_O_y_/*h*-BN_x_O_y_ (hereafter denoted as Cat) after 30 min exposure in the dark (curve 3) and after UV irradiation for 5, 15, 30, and 60 min (curves 4–7). MB + *h*-BN_x_O_y_ suspension (designated as Cat_free) was use as a control (Figure 5b). The *h*-BN_x_O_y_ sorption capacity was assessed by comparing the spectrum before and after exposure in the dark. An almost twofold decrease in intensity indicates a rather high sorption capacity of the nanomaterial.

The concentration of MB molecules is directly proportional to UV-vis absorption. The intensity of absorption spectrum of sample Cat_free does not change with UV irradiation, while sample Cat demonstrates a sequential decrease in the spectrum intensity as the duration of irradiation increases due to the MB degradation with specific catalyst mass activity of 4.58 mmol·g^−1^·h^−^^1^.

**Figure 6:**
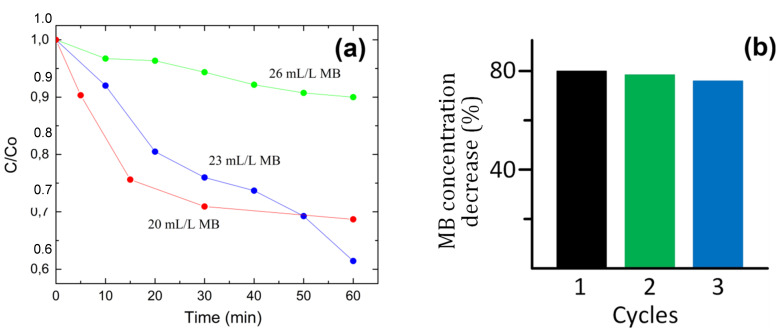
Photocatalytic performance of *a*-MoS_x_O_y_/*h*-BN_x_O_y_ catalyst upon decolorization of three concentrations of MB aqueous solutions under UV irradiation (**a**). Reusability of *a*-MoS_x_O_y_/*h*-BN_x_O_y_ catalyst during the degradation of a MB aqueous solution under UV irradiation (**b**).

To test the effect of MB concentration on its photodegradation activity, additional experiments were carried out at increased MB concentrations of 1.15 and 1.30 mL per 50 mL of water, which corresponds to molar concentrations of 71.9 and 81.3 mmol/L, respectively. The concentration of catalyst in these experiments was 6 mg per 50 mL and pH of the solution was neutral. Figure 6a shows the photocatalytic degradation of MB at the three investigated concentrations. At a MB concentration of 71.9 mmol/L (23 mL/L), MB photodegradation in the first 30 min proceeds more slowly than at a MB concentration of 62.5 mmol/L (20 mL/L). However, with further irradiation, MB decomposition proceeds faster. This may be due to the recombination of photogenerated active species, the kinetics of which depends on their concentration, and at a relatively low MB concentration, some active species can be deactivated before they reach the dye molecules. With a further increase in the MB concentration to 81.3 mmol/L, the decomposition kinetics sharply slows down. This may be due to a decrease in the intensity of UV irradiation in the solution due to the MB absorption, which has a photoabsorption band in the UV range. Thus, the highest degradation rate is observed at a MB concentration of 71.9 mmol/L, which corresponds to an activity as high as 5.51 mmol·g^−1^·h^−1^. Table 4 compares the photodegradation efficiency of *a*-MoS_x_O_y_/*h*-BN_x_O_y_ with other catalytic systems. It can be seen that the efficiency of *a*-MoS_x_O_y_/*h*-BN_x_O_y_ system is at least 4 times higher than that of other nonmetal catalysts that have been reported so far.

The reusability of the photocatalyst was evaluated from three successive photocatalytic tests during 90 min of UV irradiation of solutions with a MB concentration of 23 mL/L (71.9 mmol/L). According to the obtained results, the photocatalytic activity reaches 80 wt. % and slightly decreases after three cycles, which indicates good stability and the reusability of the material (Figure 6b).

Photocatalytic degradation of MB in water occurs due to its reaction with hydroxyl radicals (·OH), formed as a result of photochemical reactions, as described elsewhere [2]. Electron-hole pares are generated in the MoS_x_O_y_ film due to the photon absorption. Photogenerated electrons remain in MoS_x_O_y_, while holes are captured by *h*-BN_x_O_y_, which prevents electron-hole recombination (1). Electrons react with adsorbed oxygen dissolved in water and form superoxide radicals (O_2_^−^) (2). These radicals react with water to form a hydroperoxyl radical and a hydroxyl ion (3); two hydroperoxyl radicals produce hydrogen peroxide and molecular oxygen (4). Under UV irradiation, hydrogen peroxide dissociates into two hydrogen radicals (5). The hydroxyl ion is oxidized by a hole to a hydroxide radical (6). Finally, hydroxyl radicals react with MB, decomposing it into CO_2_ and H_2_O (7):

MoS_x_O_y_/*h*-BN_x_O_y_ + hν → MoS_x_O_y_(e^−^)/*h*-BN_x_O_y_(h^+^) (1)

MoS_x_O_y_(e^−^) + O_2(ads)_ → O_2_·^−^ (2)

O_2_·^−^ + H_2_O → HO_2_· + OH^−^ (3)

2HO_2_· → H_2_O_2_ + O_2_ (4)

H_2_O_2_ + hν → 2 ·OH (5)

OH^−^ + h^+^ → ·OH (6)

MB + ·OH → CO_2_ + H_2_O (7)

For the reactions described above to occur, the following conditions must be met [59]:

(8) $$E_{gap}\geq 1.23\;\mathrm{eV}$$

(9) $$E_{CB}\geq E^{0}\left(O_{2\left(ads\right)}/{\dot{O}}_{2}^{-}\right)$$

(10) $$E_{VB}\leq E^{0}\left({\mathrm{OH}}^{-}/\dot{O}H\right)$$

where *E_gap_*—is the band gap, *E_VB_* and *E_CB_*—are the positions of valence and conducting bands, respectively.

### 3.3. Theoretical Investigation

**Figure 7:**
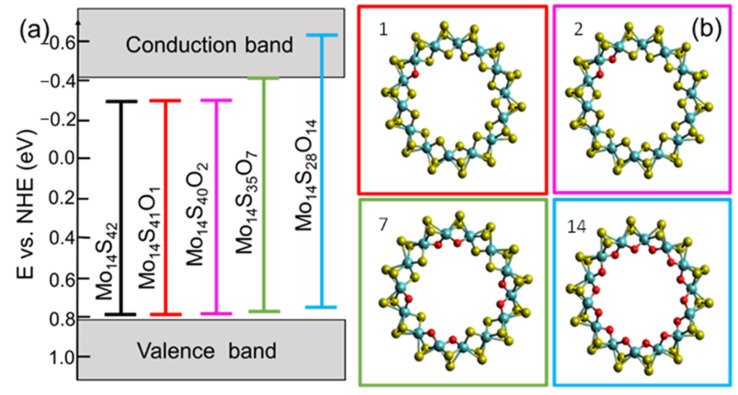
Energy diagram of the valence and conduction bands of Mo_14_S_42−X_O_X_ cluster at x = 0, 1, 2, 7, 14, in comparison with a normal hydrogen electrode (**a**) and changes in the amorphous structure of Mo_14_S_42−X_O_X_ upon oxygen substitution for sulfur (**b**). (1) x = 1, (2) x = 2, (7) x = 7, (14) x =14.

DFT analysis was performed to simulate the energy band structure and oxygen substitution level in MoS_x_O_y_, providing photoinduced water splitting. When constructing the structural model, we took into account that apical sulfur is not observed, and the MoS_3_ cluster is the most stable in the Mo–S system [60]. Therefore, the MoS_x_O_y_ structure was modeled as a chain of MoS_3_ clusters with different oxygen substitutions for sulfur. Since the main form of structure organization of amorphous molybdenum sulfide is randomly wave-linked chains consisting of MoS_3_ monomers, a structure representing a closed ring with a constant radius of curvature was chosen as a model system with a minimum energy [61]. This ring-shape MoS_3_ structure was used for further calculations. The chosen stoichiometries are also confirmed by the experimentally found oxidation states of molybdenum: Mo^+4^ and Mo^+5^. The Mo_14_S_42_ cluster was considered as isolated. For the subsequent modeling of substitutional defects, sulfur was successively replaced by oxygen. The Mo_14_S_42-X_O_X_ structure at x = 1, 2, 7, and 14 is shown in Figure 7b. Each of the presented structures corresponds to the minimum energy for a given stoichiometry.

According to Bader’s charge analysis [60], upon doping of MoS_3_ clusters, the partial charge on sulfur atoms ranges from −0.30 to −0.25, and its formal charge is −2. This charge redistribution shows that the sulfur valence electrons contribute to the Mo–S and S–S bonds, resulting in the formation of unsaturated sulfur bond.

The calculation of the band structure shows that the band gap of the isolated Mo_14_S_42_ cluster is 1.08 eV, which is insufficient for the photocatalytic splitting of water. With an increase in the fraction of impurity oxygen, the band gap increases and in the case of Mo_14_S_28_O_14_ (33%) it is 1.38 eV, which satisfies condition (8). However, in addition to the band gap, the photocatalyst must meet certain requirements for the position of the edges of the valence and conduction bands. To satisfy conditions (9) and (10), their positions must be respectively lower and higher than the potentials of the reactions catalyzed by the generated electron and hole. For each of the structures, the HOMO and LUMO values were calculated according to work [62] and correlated with the equilibrium potentials of hydrogen and oxygen evolution for pH 7. The found positions of the conduction and valence bands relative to the standard hydrogen electrode are −0.62 eV and 0.74 eV, respectively (Figure 7a).

Thus, DFT analysis shows that chained MoS_x_O_y_, consisting of MoS_3_ clusters with approximately 33% oxygen substitutions, has a band structure suitable for water photodissociation. It should be noted that MoS_x_O_y_ was modeled as a linear chain, but it may look like a branched structure with a certain number of branch points. The formation of such a chain structure can occur as a result of the reaction between MoCl_5_ and H_2_S. Five-fold coordination of Mo in MoCl_5_ promotes the formation of a chain structure. Hydroxyl groups existing on the *h*-BN_x_O_y_ surface supply oxygen to replace sulfur and form MoS_x_O_y_. When a relatively large MoS_x_O_y_ chain is formed, it is chemically adsorbed on *h*-BN_x_O_y_ and anchored through the formation of a chemical bond between the sulfur in the chain and oxygen in *h*-BN_x_O_y_. If the concentration of S–O bond is high, this leads to a redistribution of the electron density in *h*-BN_x_O_y_. This leads to a narrowing of the band gap. Figure 5c shows the UV-vis absorption spectra of the initial *h*-BN_x_O_y_ (curve 1) and *a*-MoS_x_O_y_/*h*-BN_x_O_y_ (curve 2) nanopowders suspended in methanol. The inset represents Tauc plot. It is seen that the band gap decreases from 5.25 eV (*h*-BN_x_O_y_) to 4.92 eV (*a*-MoS_x_O_y_/*h*-BN_x_O_y_).

## 4. Conclusions

A new approach to the synthesis of amorphous molybdenum sulfide based on the reaction between MoCl_5_ and H_2_S in dimethylformamide has been developed. *a*-MoS_x_O_y_/*h*-BN_x_O_y_ nanohybrids were obtained, consisting of amorphous molybdenum oxysulfide deposited on nanoflakes of hexagonal boron oxynitride (*h*-BN_x_O_y_). Based on Raman and X-ray photoelectron spectroscopy results, as well as DFT analysis, a model of the amorphous structure of MoS_x_O_y_, consisting of chains of MoS_3_ clusters with partial substitution of oxygen for sulfur, has been constructed. When oxygen is substituted, the band gap in MoS_x_O_y_ increases and at a substitution level of ~33% reaches 1.36 eV. The positions of the valence and conduction bands relative to the standard hydrogen electrode correspond to 0.74 eV and −0.62 eV. This zonal structure provides photoinduced water splitting, which is a necessary stage in the photodegradation of organic pollutants. *a*-MoS_x_O_y_/*h*-BN_x_O_y_ nanohybrids are highly active in the photocatalytic degradation of methylene blue with a specific mass activity of 5.51 mmol g^−1^ h^−1^ under illumination with a mercury lamp, which is at least four times higher than so far reported values for nonmetal catalysts. The *a*-MoS_x_O_y_/*h*-BN_x_O_y_ photocatalyst is very stable and can be reused.

## Figures and Tables

**Table 1:** Binding energy values of B 1s, N 1s, and O 1s peaks of *h*-BN_x_O_y_ and *a*-MoS_x_O_y_/*h*-BN_x_O_y_ materials in accordance with the deconvolution of XPS spectra, as shown in Figure 2d–i.

Material	Binding Energy (eV)							
	B 1s			N 1s		O 1s		
*h*-BN_x_O_y_	192.8	191.0	190.2	399.0	398.0	534.8	536.6	
*a*-MoS_x_O_y_/*h*-BNO		191.7	190.6	398.8	398.2		532.9	531.0

**Table 2:** Binding energy values of Mo 3d and S 2s peaks in accordance with the deconvolution of XPS spectrum, as shown in Figure 2j.

Binding Energy (eV)							
Mo(6+)		Mo(5+)		Mo(4+)		S 2s	
3d_3/2_	3d_5/2_	3d_3/2_	3d_5/2_	3d_3/2_	3d_5/2_		
237.9	234.8	236.3	233.1	232.9	229.8	227.0	226.1

**Table 3:** Binding energy values of S 2p peaks in accordance with the deconvolution of XPS spectrum, as shown in Figure 2k.

Binding Energy (eV)					
S 2p		S (I)		S (II)	
		2p_1/2_	2p_3/2_	2p_1/2_	2p_3/2_
170.2	168.8	164.9	162.3	165.3	163.8

**Table 4:** Photodegradation efficiency of various catalytic systems.

Catalytic System, (g L^−1^)	Dye, (g L^−1^)	Lamp	Specific Catalyst Mass Activity (mmol g^−1^ h^−1^)	Reference
MoS_2_/TiO_2_ *^)^ (0.045)	RhB (0.01)	high pressure Hg lamp, 50 W	0.766	[18]
MoS_2_/TiO_2_ *^)^ (0.045)	MO (0.01)	λ = 313 nm	1.33	[18]
Nanosheets S-BN (0.5)	RhB (0.02)	Xenon lamp, 500 W, λ > 400 nm	0.03	[32]
g-C_3_N_4_/TiO_2_ (0.04)	RhB (0.11)	Xenon arc, 350 W, λ > 420 nm	0.188	[54]
Nano (ZnO-SnO_2_) (0.2)	MB (0.02)	Hg lamp, 250 W	0.005	[55]
g-C_3_N_4_/MU (1.0)	RO16 (0.01)	Xenon lamp, 55 W, λ > 420 nm	0.009	[56]
MBTiO_2_/MoS_2_/TiO_2_	MO (0.01)	Visible light	0.014	[57]
Ag/Ag_2_O nanoparticles (0.004) with NaBH_4_	MB (~0.115)	-	~85	[58]
*a*-MoS_x_O_y_/*h*-BN_x_O_y_ **^)^ (0.006)	MB (0.02 g·L^−1^)	Hg lamp, 145 W	5.51	This work

*^)^ Calculated for the mass of MoS_2_. **^)^ Calculated for the mass of *a*-MoS_x_O_y_.

## Data Availability

The data presented in this study are available on request from the corresponding author. The data are not publicly available due to privacy.
